# Supplementary material for: Phylogeographic analysis and species distribution modelling of the wood frog Batrachyla leptopus (Batrachylidae) reveal interglacial diversification in south western Patagonia
Source: PeerJ. 2020 Oct 6;8:e9980. doi: 10.7717/peerj.9980 (PMC7546244; doi:10.7717/peerj.9980)
Supplement: Supplemental Information 1 [file peerj-08-9980-s001.docx]

**Table S1** Partitioning schemes and nucleotide substitution models for concatenated data set determined using PartitionFinder, version 2.1.1

| **Subset** | **Best Model** | **Subset Partitions** | **Subset Sites** |
| --- | --- | --- | --- |
| 1 | TVM+I+G | D-Loop | 1-687 |
| 2 | HKY+I+G | Cytb position 1, COI position 1 | 688-1327\3, 1328-1921\3 |
| 3 | GTR+G | Cytb position 2, COI position 2 | 689-1327\3, 1329-1921\3 |
| 4 | K81+I+G | Cytb position 3, POMC position 1 | 690-1327\3, 1922-2487\3 |
| 5 | TrNef+G | COI position 3 | 1330-1921\3 |
| 6 | F81+I+G | POMC position 2 | 1923-2487\3 |
| 7 | SYM+I+G | POMC position 3 | 1924-2487\3 |
| 8 | HKY+G | CRYBA1 | 2488-2776 |
