# Supplementary material for: Phylogeographic analysis and species distribution modelling of the wood frog Batrachyla leptopus (Batrachylidae) reveal interglacial diversification in south western Patagonia
Source: PeerJ. 2020 Oct 6;8:e9980. doi: 10.7717/peerj.9980 (PMC7546244; doi:10.7717/peerj.9980)
Supplement: Supplemental Information 2 [file peerj-08-9980-s002.docx]

**Table S2** Parameter estimates for the best scenario (scenario 4, Fig. 5) from the Approximate Bayesian Computation (ABC) analysis.

| **Parameter** | **Mean** | **q025** | **q975** |
| --- | --- | --- | --- |
| Ancestral *Ne* (10^5^) | 4.75 | 2.65 | 4.99 |
| *Ne*2 (10^5^) | 2.66 | 0.52 | 4.83 |
| *Ne* Lineage A (10^4^) | 5.27 | 1.85 | 8.76 |
| *Ne* Lineage B (10^4^) | 1.56 | 3.55 | 4.03 |
| *Ne* Lineage C (10^5^) | 2.56 | 1.03 | 4.64 |
| *Ne* Lineage D (10^5^) | 4.09 | 2.60 | 4.91 |
| t1 (generations x10^4^) | 2.10 | 0.69 | 3.01 |
| t2 (generations x10^4^) | 4.44 | 3.471 | 6.25 |

*Ne*: effective populations size; t: divergent time.
