# Supplementary figures and images for: Phylogeographic analysis and species distribution modelling of the wood frog Batrachyla leptopus (Batrachylidae) reveal interglacial diversification in south western Patagonia
Source: PeerJ. 2020 Oct 6;8:e9980. doi: 10.7717/peerj.9980 (PMC7546244; doi:10.7717/peerj.9980)

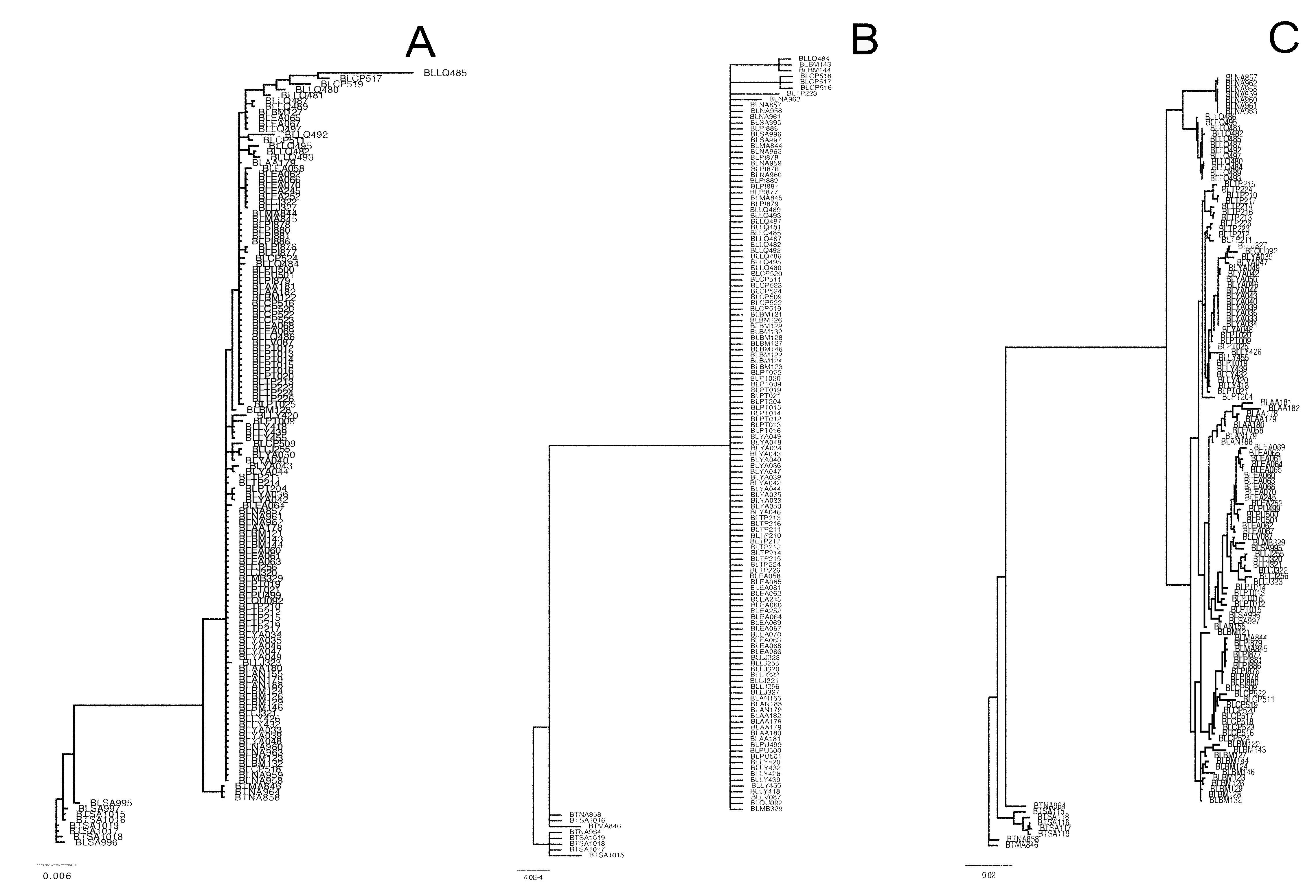

Supplement: Supplemental Information 3 — Bayesian reconstruction of sequence data of B. leptopus populations. A: Mitochondrial DNA (d-loop, cytb, COI), B: pomc: C: CrybA1. [file peerj-08-9980-s003.png]
